# Improving the Fermentation Production of the Individual Key Triterpene Ganoderic Acid Me by the Medicinal Fungus *Ganoderma lucidum* in Submerged Culture

**DOI:** 10.3390/molecules171112575

**Published:** 2012-10-24

**Authors:** Gao-Qiang Liu, Xiao-Ling Wang, Wen-Jun Han, Qin-Lu Lin

**Affiliations:** 1 Hunan Provincial Key Laboratory of Forestry Biotechnology, College of Life Science and Technology, Central South University of Forestry and Technology, Changsha 410004, China; Email: wxl_edu@yahoo.com.cn (X.-L.W.); qinxinhuzhu@tom.com (W.-J.H.); 2 National Engineering Laboratory for Rice and By-product Further Processing, Central South University of Forestry and Technology, Changsha 410004, China; Email: phdqlin@yahoo.cn

**Keywords:** medicinal fungi, *Ganoderma lucidum*, submerged culture, response surface methodology, ganoderic acid Me

## Abstract

Enhanced ganoderic acid Me (GA-Me, an important anti-tumor triterpene) yield was attained with the medicinal fungus *Ganoderma lucidum* using response surface methodology (RSM). Interactions were studied with three variables, viz. glucose, peptone and culture time using a Central Composite Design (CCD). The CCD contains a total of 20 experiments with the first 14 experiments organized in a fractional factorial design, with the experimental trails from 15 to 20 involving the replications of the central points. A polynomial model, describing the relationships between the yield of GA-Me and the three factors in a second-order equation, was developed. The model predicted the maximum GA-Me yield of 11.9 mg·L^−1^ for glucose, peptone, culture time values of 44.4 g·L^−1^, 5.0 g·L^−1^, 437.1 h, respectively, and a maximum GA-Me yield of 12.4 mg·L^−1^ was obtained in the validation experiment, which represented a 129.6% increase in titre compared to that of the non-optimized conditions. In addition, 11.4 mg·L^−1^ of GA-Me was obtained in a 30-L agitated fermenter under the optimized conditions, suggesting the submerged culture conditions optimized in the present study were also suitable for GA-Me production on a large scale.

## 1. Introduction

*Ganoderma lucidum* (Leyss. ex Fr.) Karst (“Ling-zhi” in Chinese and “Reishi” in Japanese) is a famous medicinal mushroom that has been used as a traditional medicine for a long time in Asia [1]. The potential medicinal value and wide acceptability of this edible mushroom have attracted intense interest in the search for its biologically active substances during the last 30 years [2,3,4]. Nowadays, *G. lucidum* and related products are widely used not only as health foods, but also clinical drugs for the prevention and treatment of hepatopathy, chronic hepatitis, nephritis, gastric ulcer, hypertension, arthritis, neurasthenia, insomnia, asthma, acute and chronic bronchitis, leucopoenia, and cancer [5,6]. 

Modern chemistry studies show that *G. lucidum* contains a variety of phytochemicals. Some of the potent biologically active compounds that have been shown to possess diverse and potentially significant pharmacological activities are the bitter triterpenes (especially ganoderic acids, GAs) [7,8,9,10]. Since the first discovery of ganoderic acids A and B (GA-A, GA-B), over 150 types of triterpenes have been isolated from various parts of *G. lucidum* [11], among which GA-Me has received considerable attention due to its conspicuous pharmacological properties, especially anticancer activity. GA-Me effectively inhibited tumor growth, lung metastasis [12], and tumor invasion through down-regulating matrix metalloproteinase 2/9 (MMP2/9) gene expression [13]. It was found that GA-Me depressed the viability of tumor cells at much lower concentrations than against normal cells [14], making it an effective potential therapeutic drug for the prevention and treatment of tumors in the clinic. In addition, GA-Me was demonstrated to inhibit cholesterol synthesis [10,15].

Currently, submerged fermentation of *G. lucidum* is viewed as a promising alternative for efficient production of GAs because it usually takes several months to cultivate the fruiting body of *G. lucidum* and it is also difficult to control the product quality during its cultivation [16,17]. Many researchers have focused on studying fermentation conditions to accelerate mycelial growth and optimize the production of GA by mycelia fermentation [16,17,18,19]. However, most of those previous reports were about total crude GAs production based on UV absorbance measurements [16,17,18,19], and the total GAs are a complicated mixture which usually also contains other organic acids [20] so the content may not accurately reflect GA accumulation. In addition, not all the GAs are bioactive. Therefore, investigation of the individual key GA production in submerged culture is necessary. In our previous study, we established the key factors (glucose, peptone and culture time) for total crude GA production in submerged culture [17]. The aim of this work was to improve the fermentation production of individual GA-Me based on the three key factors and study the effects of the mutual interactions of these factors on individual GA-Me production using a central composite design (CCD) and response surface methodology (RSM).

## 2. Results and Discussion

### 2.1. Production of GA-Me under Non-Optimized Culture Conditions

According to the culture media and fermentation conditions used for the maximum production of total crude GAs in our previous work [17] (as described in the Experimental section: basic fermentation conditions), triplicate experiments were carried out to examine the individual GA-Me production yield. The mycelial dry weight (DW) reached 13.1 g·L^−1^, and a 5.4 mg·L^−1^ of GA-Me production yield was obtained in *G. lucidum* cultures, of which, 2.8 mg·L^−1^ was obtained in the mycelia and 2.6 mg·L^−1^ was collected from the fermented supernatant (after removal of mycelia by centrifugation). The content of GA-Me of mycelia was 21.3 μg·100 mg^−1^ DW.

### 2.2. Optimization of GA-Me Production by CCD and RSM

In our earlier study, glucose, peptone and culture time were the key factors for GA production [17]. In the present work, the levels of the three key factors were further optimized by CCD and RSM to improve the production yield of individual GA-Me.

#### 2.2.1. Response Surface Analysis for the Optimization of Culture Condition Levels

A central composite design (CCD) was used in the optimization of GA-Me production. The ranges and the levels of the variables investigated in this study are given in Table 1.

**Table 1:** Experimental range and levels of the independent variables of ganoderic acid Me (GA-Me) optimization.

Independent variables	Range and levels				
	−1.682	−1	0	1	1.682
Glucose, *x*_1_ (g·L^−1^)	36.59	40.0	45.0	50.0	53.41
Peptone, *x*_2_ (g·L^−1^)	3.32	4.00	5.0	6.00	6.68
Fermentation time, *x*_3_ (h)	265.91	300	13	400	434.09

The results of CCD experiments for GA-Me production are presented in Table 2. Table 3 shows the analysis of variance for the experiment. The Fisher’s F-test with a very low probability value [(*P*_model_ > *F*) = 0.0002] for the total model indicated the model was highly significant. The goodness of fit of the model was examined by the coefficient of determination (*R*^2^ = 0.9257), which implied that more than 92% of the sample variation was attributable to the variables and only 7.43% of the total variance could not be explained by the model. The adjusted determination coefficient (Adj. *R^2^* = 0.8570) was also satisfactory to confirm the significance of the model. 

Table 4 reveals the analysis of variance (ANOVA) for three key factors on the production of GA-Me. The smaller the *P*-value, the more significant is the corresponding factor. *X*_3_ (culture time) had extremely significant effect on GA-Me production (*P* < 0.01), followed by *x*_2_ (peptone concentration), suggesting culture time and peptone concentration are very important for GA-Me production in submerged fermentation of *G. lucidum*.

**Table 2:** The central composite design matrix and the response of ganoderic acid Me (GA-Me) of *G. lucidum*.

Runs	Coded values			*Y_GA-Me_* (mg·L^−1^)
	*Factor x*_1_	*Factor x*_2_	*Factor x*_3_	
1	−1	−1	−1	4.9
2	−1	−1	1	9.9
3	−1	1	−1	4.1
4	−1	1	1	7.4
5	1	−1	−1	6.9
6	1	−1	1	10.5
7	1	1	−1	5.1
8	1	1	1	6.7
9	−1.682	0	0	6.1
10	1.682	0	0	8.1
11	0	−1.682	0	7.0
12	0	1.682	0	6.7
13	0	0	−1.682	3.1
14	0	0	1.682	11.2
15	0	0	0	8.9
16	0	0	0	8.8
17	0	0	0	8.8
18	0	0	0	8.5
19	0	0	0	7.9
20	0	0	0	8.2

**Table 3:** Analysis of variance (ANOVA) for the full quadratic model for optimization of ganoderic acid Me (GA-Me) production in *G. lucidum*.

Regression	DF	Sum of Squares	*R*-Square	*F* value	*Pr* > *F*
Linear	3	63.2143	0.7614	34.16	<0.0001 **
Quadratic	3	10.0708	0.1213	5.44	0.0177 *
Crossproduct	3	3.5738	0.0430	1.93	0.1886
Total model	9	76.8589	0.9257	13.84	0.0002 **

*R^2^* *=* 0.9257; Adj. *R^2^ =* 0.8570; ** Significant at 0.01 level, * Significant at 0.05 level.

**Table 4:** Analysis of variance (ANOVA) for three factors for optimization of ganoderic acid Me (GA-Me) production of *G. lucidum*.

Factor	DF	Sum of Squares	Mean Square	*F* value	*Pr* > F
x1	4	8.3684	2.0921	3.39	0.0534
x2	4	13.8737	3.4684	5.62	0.0123 *
x3	4	60.1597	15.0399	24.38	<0.0001 **

** Significant at 0.01 level, * Significant at 0.05 level.

The significance of each coefficient was determined by Student’s *t*-test and *P*-value, which is listed in Table 5 The larger the magnitude of *t*-test and smaller the *P*-value, the more significant is the corresponding coefficient. The polynomial model for GA-Me yield *Y*_GA-Me_ was regressed by all the terms and was expressed by Equation (1). The regression equation showed that the GA-Me yield was an empirical function of test variables in coded unit. 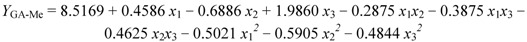
(1)
where *Y*_GA-Me_ is the predicted GA-Me yield, *x*_1_ glucose, *x*_2_ peptone, and *x*_3_ is culture time.

**Table 5:** Results of regression analysis of a predictive polynomial model for optimization of ganoderic acid Me (GA-Me) of *G. lucidum*.

Parameter	DF	Coefficients estimated	Standard error	*t* value	*Pr* > |*t*|
Intercept	1	8.5169	0.3203	26.59	<0.0001 **
*x*_1_	1	0.4586	0.2125	2.16	0.0563
*x*_2_	1	−0.6886	0.2125	−3.24	0.0089 **
*x*_3_	1	1.9860	0.2125	9.34	<0.0001 **
*x*_1_*x*_1_	1	−0.5021	0.2069	−2.43	0.0356 *
*x*_1_*x*_2_	1	−0.2875	0.2777	−1.04	0.3249
*x*_2_*x*_2_	1	−0.5905	0.2069	−2.85	0.0171 *
*x*_1_*x*_3_	1	−0.3875	0.2777	−1.40	0.1931
*x*_2_*x*_3_	1	−0.4625	0.2777	−1.67	0.1268
*x*_3_*x*_3_	1	−0.4844	0.2069	−2.34	0.0412 *

** Significant at 0.01 level, * Significant at 0.05 level.

Equation (1) also reveals that culture time (*x*_3_) had a strong positive linear effect on the response (*P* < 0.01) of *Y*_GA-Me_ as it had the largest coefficient, followed by peptone (*x*_2_), which showed a significant negative linear effect (*P* < 0.01). However, glucose (*x*_1_) had no significant effect on GA-Me production at the tested concentrations (*P* > 0.05), and the above three variables also indicated negative quadric effects on GA-Me yield (*P* < 0.05). No significant interactions were noted between any two of the three variables (*P* > 0.05).

#### 2.2.2. Interaction among the Factors and Selection of Their Optimum Levels

**Figure 1:**
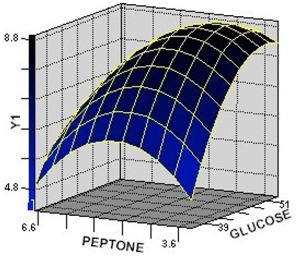
The surface plot of the combined effects of glucose and peptone on Y_1_ (GA-Me) production. Fixed level: culture time = 0 (coded value).

**Figure 2:**
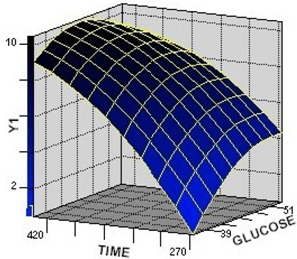
The surface plot of the combined effects of glucose and culture time on Y_1_ (GA-Me) production by *G. lucidum*. Fixed level: peptone concentration = 0 (coded value).

**Figure 3:**
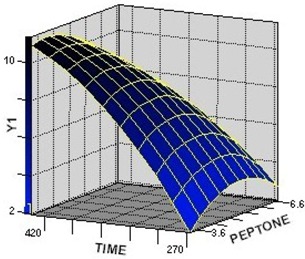
The surface plot of the combined effects of peptone and culture time on Y_1_ (GA-Me) production by *G. lucidum*. Fixed level: glucose concentration = 0 (coded value).

The 3D response surface is generally the graphical representation of the regression equation. Figure 1, Figure 2, Figure 3 represent the 3D response surfaces for the optimization of culture condtion of GA-Me production. Each figure presented the effect of two variables on the production of GA-Me, while another variable was held at zero level (coded value).

From the response surface plots, it is easy to understand the interactions between two nutrients and also to locate their optimum levels. It can be seen from Figure 1 and Figure 2 that the yield of GA-Me was high when the concentrations of peptone were in the range of 3.6–4.8 g·L^−1^ and glucose in the range of 45–51 g·L^−1^ synchronously. Figure 2 and Figure 3 showed that GA-Me yield increased upon increasing the culture time from 270 to 420 h. The model predicted a maximum GA-Me yield of 11.9 mg·L^−1^ for glucose, peptone, culture time values of 44.4 g·L^−1^, 5.0 g·L^−1^, 437.1 h, respectively.

#### 2.2.3. Verification of the Models in Flask Culture and Bioreactor Culture

Triplicate experiments were carried out to verify the availability and accuracy of the model (Equation (1)) for GA-Me production in flask shake culture. The results are shown in Table 6. Under the calculated optimum culture conditions, a cell density of 11.3 g·L^−1^ (by dry weight, DW) was obtained, and the content of GA-Me in mycelial cell was 42.7 μg·100 mg^−1^ DW (also for 4.8 mg·L^−1^ based on 11.3 g·L^−1^ of mycelia), and that in fermented supernatant (removal of mycelia by centrifugation) it was 7.6 mg·L^−1^, that is, a 12.4 mg·L^−1^ of GA-Me production yield was obtained in *G. lucidum* cultures, which represented a 129.6% increase in titre compared to the the non-optimized culture conditions (5.4 mg·L^−1^), and was also in agreement with the predicted value (11.9 mg·L^−1^). The good agreement between the predicted and experimental results verifies the validity of the model and the existence of an optimum point. The feasibility of the regression model was further tested in a 30-L scaled fermenter under the optimized culture condition. A maximum yield (11.4 mg·L^−1^) of GA-Me was obtained (Table 6), suggesting the culture condition optimized in the present study was also suitable for GA-Me production in a large scale.

**Table 6:** Verification results for ganoderic acid Me (GA-Me) production under the optimized culture conditionby *G. lucidum*.

Culture systems	Cell density (g·L^−1^)	GA-Me content in Cell (μg/100 mg DW)	GA-Me production in Cell (mg·L^−1^)	GA-Me production in fermented broth (mg·L^−1^)	Final glucose concentration (g·L^−1^)
Flask culture	11.3 ± 0.2	42.7 ± 0.4	4.8 ± 0.7	7.6 ± 0.2	4.7 ± 0.3
Bioreactor culture	9.1 ± 0.5	33.9 ± 0.6	3.1 ± 0.2	8.3 ± 0.7	4.3 ± 0.6

Until now, GAs were mainly extracted from the solid cultivated fruiting bodies of *G. lucidum* [21]. It usually takes several months to cultivate the fruiting body, and the yield of GAs is very low [11]. Consequently, cell culture of *G. lucidum* is recognized as a promising alternative for efficient production of GAs [17,18,19]. Although it has been reported that GA accumulation was influenced by the culture medium and process conditions of *G. lucidum*, many studies were about total GAs production [16,17,18,19], and there are few reports available for individual GAs. Xu *et al.* studied the production of individual ganoderic acids (GA-Me, GA-T, GA-S and GA-Mk) by *G. lucidum* cell culture [22]. It was found that GA-Me yield in liquid static culture was higher than that obtained in liquid shaking culture. The GA-Me content in static culture was in the range of 9.6–11.5 mg·L^−1^ (cell density: 10–12.5 g·L^−1^; GA-Me content: 96 μg·100 mg^−1^ DW), and the GA-Me content was higher than that obtained in the present study. However, the GA-Me yield was very low in shaking flask culture with a lactose-based culture medium, and the content of GA-Me in the mycelium was about 10.1 μg·100 mg^−1^ DW, which is lower than that in the present work. Nevertheless the content of GA-Me in fermented supernatant (extracellular GA-Me) was not determined by Xu *et al*. In the present study, we obtained 7.6 mg·L^−1^ of GA-Me from fermented supernatant, and 4.8 mg·L^−1^ of GA-Me from mycelia, suggesting some quantity of GA-Me was excreted into the fermented broth during the culture process, a phenomenon also observed in several previous studies [17,21,22,23,24,25]. In addition, the optimized culture condition was successfully applied in GA-Me production in an agitated 30-L fermenter. Generally, in submerged mushroom cultures, it is necessary to agitate the culture broth in order to obtain good mixing and thereby promote heat and mass transfer [26]. However, agitation also creates shear force. The shear stress exerted by the impeller blades in an agitated fermenter usually reduces and modifies the mycelium pellet growth in terms of diameter, circularity and compactness, and the accumulation of metabolites. In this work, the mycelium showed no significant damage caused by the shear forces with a speed of 150 rpm·min^−1^, and 9.1 g·L^−1^ of mycelium pellets and a 11.4 mg·L^−1^ titre of GA-Me was obtained, suggesting GA-Me could be also produced under the optimized culture conditions in a large-scale agitated bioreactor.

## 3. Experimental

### 3.1. Microorganism

Stock cultures of *G. ludicum* SCIM 0006 were obtained from the Strain Collection of Industrial Microorganisms (SCIM), Central South University of Forestry & Technology (Changsha, China), and maintained on potato-dextrose-agar slants, stored at 4 °C.

### 3.2. Liquid Shake Culture

*G. lucidum* SCIM 0006 was grown in a 250-mL flask containing 80 mL seed culture medium (see below) at 30 °C for 8 days with shaking at 160 rpm. This was then inoculated at 12% (v/v) into the fermentation medium incubated at 28 °C for different days for optimization of GA-Me production. The seed culture medium composed of (in g·L^−1^): glucose (40.0), peptone (4.0), KH_2_PO_4_ (0.75), MgSO_4_·7H_2_O (0.45) and vitamin B_1_ (0.01). The fermentation medium composed of (g·L^−1^): glucose (36.595–3.41), peptone (3.326–68), KH_2_PO_4_ (0.75), MgSO_4_·7H_2_O (0.50) and vitamin B_1_ (0.01). The basic fermentation conditions were as follows: glucose (38 g·L^−1^), peptone (4.5 g·L^−1^), KH_2_PO_4_ (0.75), MgSO_4_·7H_2_O (0.45) and vitamin B_1_ (0.01); culture time 300 h; culture temperature 28 °C [17].

### 3.3. Bioreactor Culture

The bioreactor culture was carried out by a similar method described in our previous work [23] in an agitated 30-L fermenter (New Brunswick Scientific Co., Enfield, CT, USA), under the following conditions: medium volume 20 L, inoculation volume 12% (v/v), temperature 28 °C, aeration rate 6.0 vvm, and agitation speed 150 rpm.

### 3.4. Determination of Dry Cell Weight

Samples collected from flasks were filtered using a 40-mesh stainless sieve and the mycelium was harvested. Mycelial biomass was collected by centrifuging the mycelium at 8,000 rpm for 15 min, washing the precipitated cells for three times with distilled water, and drying at 60 °C until it reached to a constant weight [23].

### 3.5. Measurement of GA-Me

The GA-Me yield (mg·L^−1^) includes the contents of GA-Me from both mycylia and fermented broth. The determination of GA-Me was made by a similar method described in a previous work [22]. The dried mycelia (2 g) were extracted by circumfluence with 70% (v/v) ethanol (100 mL) for 2 h (twice), and ultrasonic treatment for 1 h (three times). The supernatant was dried at 50 °C under vacuum, the residues were suspended in water, and redissolved in absolute ethanol for high performance liquid chromatography (HPLC) analysis. For treatment of GA-Me samples from fermented broth, after removal of mycelia by centrifugation, the supernatant was dried at 50 °C under vacuum, and the residues were suspended in water, and redissolved in absolute ethanol for HPLC analysis. The analysis was perfomed on a Agilent 1200 series HPLC instrument (5 μm Agilent Zorbax SB-C18 column, 250 × 4.6 mm). The elution was performed at a flow rate of 1.0 mL·min^−1^ with a linear gradient of solvent A (methanol/acetic acid, 100:0.5, v/v) and solvent B (water). The gradient, starting at sample injection, was linear from 80% to 100% A in 20 min and the elution continued for an additional 20 min at 100% A. The sample injection volume was 20 μL, GA-Me was detected at 245 nm. Standard curves of peak area, as a function of the concentration of GA-Me was prepared for quantitative analysis [22]. The standard of GA-Me was extracted and purified from mycelia with preparative liquid chromatography in our lab with purity over 96%.

### 3.6. RSM Experimental Design and Statistical Analysis

The preliminary trails indicated that glucose, peptone and fermentation time were the significant variables for GA production. Hence, these three variables were chosen to obtain the optimum levels. A central composite design (CCD) was used in the optimization of GA-Me production. Table 1 shows the ranges and the levels of the variables. The lowest and the highest levels of variables were: glucose, 36.59 and 53.41 g·L^−1^; peptone 3.32 and 6.68 g·L^−1^; time, 265.91 and 434.09 h. All variables were taken at a central coded value considered as zero. The CCD contains a total of 20 experiments with the first 14 experiments organized in a fractional factorial design, with the experimental trails from 15 to 20 involving the replications of the central points (Table 2). A mathematical model, describing the relationships between the process indice (the yield of GA-Me) and the fermentation parameters in second-order equation, was developed. The yield of GA-Me by *G. lucidum* was multiply regressed with respect to the fermentation parameters by the least squares method (Equation (2)) as follows:

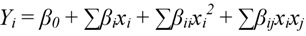
(2)
where *Y_i_* is the predicted response variable; *β_0_*, *β_i_*, *β_ii_*, *β_ij_* are constant regression coefficients of the model, and *x_i_*, *x_j_* (*i* = 1, 3; *j* = 1, 3, *I* ≠ *j*) represent the independent variables (fermentation parameters) in the form of coded values.

The accuracy and general ability of the above polynomial model could be evaluated by the coefficient of determination *R*^2^. The significance of each coefficient was determined using Student’s t-test.

The SAS statistical package [27] was used for regression and graphical analysis of data obtained. The optimum levels of glucose, peptone and fermentation time were obtained by solving the regression equation.

## 4. Conclusions

Enhanced production of individual GA-Me, an important bioactive triterpene, by *G. lucidum* in submerged culture was studied. From the present study, it is evident that the use of a statistical culture condition optimization approach and response surface methodology was helpful to locate the optimum levels of the significant conditions with minimum effort and time. The optimization of the culture conditions not only resulted in a 129.6% higher GA-Me concentration than using non-optimized medium, but also in a successful large scale fermentation process. In addition, GA-Me is not only present in *G. lucidum* mycelia but also in fermented broth.

## References

[1] Liu G.Q., Zhang K.C. (2005). Mechanisms of the anticancer action of *Ganoderma lucidum* (Leyss. ex. Fr.) Karst.: A new understanding. J. Integr. Plant Biol..

[2] Cheung W.M.W., Hui W.S., Chu P.W.K., Chiu S.W., Ip N.Y. (2000). *Ganoderma* extract activates MAP kinases and induces the neuronal differentiation of rat pheochromocytoma PC12 cells. FEBS Lett..

[3] Gao J.J., Min B.S., Ahn E.M., Nakamura N., Lee H.K., Hattori M. (2002). New triterpene aldehydes, lucialdehydes A–C, from *Ganoderma lucidum* and their cytotoxicity against murine and human tumor cells. Chem. Pharm. Bull..

[4] Ha T.B.T., Gerhauser C., Zhang W.D., Ho-Chong-Line N., Fouraste I. (2000). New lanostanoids from *Ganoderma lucidum* that induce NAD(P)H: Quinone oxidoreductase in cultured hepalclc7 murine hepatoma cells. Planta Med..

[5] Gao Y.H., Zhou S.F., Jiang W.Q., Huang M., Dai X.H. (2003). Effects of Ganopoly(R) (a *Ganoderma lucidum* polysaccharide extract) on the immune functions in advanced-stage cancer patients. Immunol. Invest..

[6] Miyamoto I., Liu J., Shimizu K., Sato M., Kukita A., Kukita T., Kondo R. (2009). Regulation of osteoclastogenesis by ganoderic acid DM isolated from *Ganoderma lucidum*. Eur. J. Pharmacol..

[7] Chen N.H., Liu J.W., Zhong J.J. (2010). Ganoderic acid T inhibits tumor invasion *in vitro* and *in vivo* through inhibition of MMP expression. Pharmacol. Rep..

[8] Chen Y.S., Ming S.S., Cheng T.W. (1999). Differential effects of ganodermic acid S on the thromboxane A2-signaling pathways in human platelets. Biochem. Pharmacol..

[9] Kimura Y., Taniguchi M., Baba K. (2002). Antitumor and antimetastatic effects on liver of triterpenoid fractions of *Ganoderma lucidum*: Mechanism of action and isolation of an active substance. Anticancer Res..

[10] Min B.S., Gao J.J., Nakamura N., Hattori M. (2000). Triterpenes from the spores of *Ganoderma lucidum* and their cytotoxicity against meth-A and LLC tumor cells. Chem. Pharm. Bull..

[11] Xu J.W., Zhao W., Zhong J.J. (2010). Biotechnological production and application of ganoderic acids. Appl. Microbiol. Biotechnol..

[12] Wang G., Zhao J., Liu J.W., Huang Y., Zhong J.J., Tang W. (2007). Enhancement of IL-2 and IFN-γ expression and NK cells activity involved in the anti-tumor effect of ganoderic acid Me *in vivo*. Int. Immunopharmacol..

[13] Chen N.H., Liu J.W., Zhong J.J. (2007). Ganoderic acid Me inhibits tumor invasion through down-regulating matrix metalloproteinases 2/9 gene expression. J. Pharmacol. Sci..

[14] Chen N.H., Zhong J.J. (2009). Ganoderic acid Me induces G1 arrest in wild-type p53 human tumor cells while G1/S transition arrest in p53-null cells. Process Biochem..

[15] Komoda Y., Shimizu M., Sonoda Y., Sato Y. (1989). Ganoderic acid and derivatives as cholesterol synthesis inhibitors. Chem. Pharm. Bull..

[16] Fang Q.H., Zhong J.J. (2002). Two-stage culture process for improved production of ganoderic acid by liquid fermentation of higher fungus *Ganoderman lucidum*. Biotechnol. Progr..

[17] Liu G.Q., Xiao H.X., Wang X.L., Zhao Y., Zhang Y.G., Ren G.P. (2011). Stimulated production of triterpenoids of *Ganoderma lucidum* by an ether extract from the medicinal insect, *Catharsius molossu*s and identification of the key stimulating active components. Appl. Biochem. Biotechnol..

[18] Tang Y.J., Zhang W., Zhong J.J. (2009). Performance analyses of a pH-shift and DOT-shift integrated fed-batch fermentation process for the production of ganoderic acid and *Ganoderma* polysaccharides by medicinal mushroom *Ganoderma lucidum*. Bioresource Technol..

[19] Zhang W.X., Zhong J.J. (2010). Effect of oxygen concentration in gas phase on sporulation and individual ganoderic acids accumulation in liquid static culture of *Ganoderma lucidum*. J.Biosci. Bioeng..

[20] Wagner R., Mitchell D.A., Sassaki G.L., De Almeida Amazonas M.A.L., Berovic M. (2003). Current techniques for the cultivation of *Ganoderma lucidum* for the production of biomass, ganoderic acid and polysaccharides. Food Technol. Biotechnol..

[21] Xu P., Ding Z.Y., Qian Z., Zhao C.X., Zhang K.C. (2008). Improved production of mycelial biomass and ganoderic acid by submerged culture of *Ganoderma lucidum* SB97 using complex media. Enzyme Microb. Technol..

[22] Xu J.W., Xu Y.N., Zhong J.J. (2010). Production of individual ganoderic acids and expression of biosynthetic genes in liquid static and shaking cultures of *Ganoderma lucidum*. Appl. Microbiol. Biotechnol..

[23] Liu G.Q., Wang X.L. (2007). Optimization of critical medium components using response surface methodology for biomass and extracellular polysaccharide production by *Agaricus blazei*. Appl. Microbiol. Biotechnol..

[24] Liu G.Q., Ding C.Y., Zhang K.C. (2008). Effects of powdered dung beetle (*Catharsius molossus*) on cell growth and triterpenoid production of *Ganoderma lucidum*. Mycosystema.

[25] Baskar G., Rajesh L.K.S., Pavithra S.K., Aarathi S., Renganathan S. (2011). Production of ganoderic acid by *Ganoderma lucidium* MTCC 1039 from cottonseed oil cake, Statistical screening of process variables. Indian J. Biotechnol..

[26] Gong H.G., Zhong J.J. (2005). Hydrodynamic shear stress affects cell growth and metabolite production by medicinal mushroom *Ganoderma lucidum*. Chin. J.Chem. Eng..

[27] (2000). The SAS Statistical Package, version 8.1; an integrated system of software;.

